# Re-positive testing, clinical evolution and clearance of infection: results from COVID-19 cases in isolation in Viet Nam

**DOI:** 10.5365/wpsar.2021.12.4.857

**Published:** 2021-12-13

**Authors:** Ngoc-Anh Hoang, Thai Quang Pham, Ha-Linh Quach, Khanh Cong Nguyen, Samantha Colquhoun, Stephen Lambert, Duong Huy Luong, Quang Dai Tran, Dinh Cong Phung, Tran Nhu Duong, Nghia Duy Ngu, Tu Anh Tran, Hue Bich Thi Nguyen, Duc-Anh Dang, Florian Vogt

**Affiliations:** a Department of Epidemiology, National Institute of Hygiene and Epidemiology, Hanoi, Viet Nam.; b National Centre for Epidemiology and Population Health, Research School of Population Health, College of Health and Medicine, Australian National University, Canberra, Australia.; c School of Preventive Medicine and Public Health, Hanoi Medical University, Hanoi, Viet Nam.; d General Department of Preventive Medicine, Ministry of Health, Hanoi, Viet Nam.; e National Agency for Science and Technology Information, Ministry of Science and Technology, Viet Nam.; f National Institute of Hygiene and Epidemiology, Hanoi, Viet Nam.; g National Hospital of Tropical Diseases, Hanoi, Viet Nam.; h The Kirby Institute, University of New South Wales, Sydney, NSW, Australia.; * These authors contributed equally.; # These authors contributed equally.

## Abstract

**Objective:**

Asymptomatic infection with severe acute respiratory syndrome coronavirus 2 (SARS-CoV-2) and test re-positivity after a negative test have raised concerns about the ability to effectively control the coronavirus disease 2019 (COVID-19) pandemic. We aimed to investigate the prevalence of COVID-19 asymptomatic and pre-symptomatic infections during the second wave of COVID-19 in Viet Nam, and to better understand the duration of SARS-CoV-2 infection and the dynamics between the evolution of clinical symptoms and SARS-CoV-2 test positivity among confirmed COVID-19 cases.

**Methods:**

We conducted a cohort analysis on the first 50 confirmed cases during the second COVID-19 wave in Viet Nam using clinical, laboratory and epidemiological data collected from 9 March to 30 April 2020. Kaplan–Meier estimates were used to assess time to clearance of SARS-CoV-2 infection, and log-rank tests were used to explore factors related to time to SARS-CoV-2 infection clearance.

**Results:**

Most cases (58%) had no typical signs or symptoms of COVID-19 at the time of diagnosis. Ten cases (20%) were re-positive for SARS-CoV-2 during infection. Eight cases (16%) experienced COVID-19 symptoms after testing negative for SARS-CoV-2. The median duration from symptom onset until clearance of infection was 14 days (range: 6–31); it was longer in re-positive and older patients and those with pre-existing conditions.

**Conclusion:**

Asymptomatic and pre-symptomatic infections were common during the second wave of COVID-19 in Viet Nam. Re-positivity was frequent during hospitalization and led to a long duration of SARS-CoV-2 infection.

Coronavirus disease 2019 (COVID-19) is a respiratory disease caused by infection with severe acute respiratory syndrome coronavirus 2 (SARS-CoV-2). The COVID-19 pandemic, first reported in Wuhan, China in December 2019, (*1*) spread quickly worldwide. As of mid-October 2021, there have been over 240 million confirmed cases and 4.9 million deaths. (*2*) The first case of COVID-19 in Viet Nam was recorded on 22 January 2020 in a person who had returned from Wuhan, and that case was linked to a further 15 cases related to Wuhan. (*3*) By the end of February, all 16 cases had recovered and Viet Nam remained clear of COVID-19 for the following 20 days. By early March, the world saw a major shift in the distribution of COVID-19 cases from China to Europe and the United States of America, while China’s incidence decreased. (*4*) This sparked a second wave of imported COVID-19 cases in Viet Nam, of non-Chinese origin, starting on 6 March 2020 when an international passenger arriving from the United Kingdom of Great Britain and Northern Ireland tested positive. (*5*)

Case investigations conducted during the second wave suggested the occurrence of cases without compatible signs or symptoms of COVID-19 at the time of the first positive test, raising concerns about the community spread of COVID-19 in Viet Nam. Some cases remained asymptomatic until discharge, whereas others developed symptom onset after testing positive (pre-symptomatic infections). Also seen at that time was reversion of test results in patients who had tested negative following a positive result, and then returned to positive (re-positivity). As in other settings, pre-symptomatic and fully asymptomatic infections were also recorded but not systematically investigated. (*6*, *7*)

Important evidence gaps remain for asymptomatic and pre-symptomatic cases, and for patients with re-positive test results. (*8*, *9*) In particular, the duration until clearance of infection and the dynamics between clinical symptoms and test positivity are poorly understood. (*10*, *11*) The testing and quarantine policy during the initial phase of the second wave of infections in Viet Nam provided us with a setting to investigate these questions. Using clinical, laboratory and epidemiological data of arriving air travel passengers to Viet Nam and their secondary cases during March and April 2020, we aimed to investigate the prevalence of asymptomatic and pre-symptomatic COVID-19 infections and to better understand the duration of SARS-CoV-2 infection and the dynamics between the evolution of clinical symptoms and SARS-CoV-2 test positivity.

## Methods

### Design

A cohort analysis was conducted on the first 50 laboratory-confirmed cases during the second COVID-19 wave in Viet Nam using clinical, laboratory and epidemiological data collected as a part of the national epidemic response between 9 March and 30 April 2020.

### Data sources

In Viet Nam, all hospitals reported clinical and treatment information and test results for COVID-19 cases to the National Institute of Hygiene and Epidemiology and the Medical Services Administration. Data included in this analysis were obtained from the National Institute of Hygiene and Epidemiology.

### Case classification and definitions

This study used case definitions from guidelines developed by the Viet Nam Ministry of Health. (*12*) Case confirmation required a positive polymerase chain reaction (PCR) test for SARS-CoV-2. A symptomatic COVID-19 case was defined as a confirmed case showing any COVID-19 compatible symptom according to Ministry of Health guidelines, including cough, fever, muscle soreness, shortness of breath, sore throat, headache, nausea and fatigue with symptom onset within 14 days before the first positive PCR test result. (*12*) An *asymptomatic* case was a confirmed case without COVID-19 compatible symptoms throughout the incubation and infection period. This period was counted from 14 days before the first SARS-CoV-2 positive test result until the first negative PCR test, in a series of three negative PCR tests, with at least 24 hours between each test. A *pre-symptomatic* case was defined as a confirmed case without COVID-19 compatible symptoms at the time of the first positive PCR test but who then developed symptoms during the course of infection. A *re-positive* case was defined as a patient who had tested positive, then negative and then returned to positive.

A close contact was defined as a person with direct contact (£2 m distance) with a confirmed case. (*13*) In Viet Nam, if the confirmed case had a flight travel history within 14 days from the date of symptom onset or date of confirmation, whichever came first, all passengers on those flights were categorized as close contacts and were tested for SARS-CoV-2 infection.

As per Ministry of Health guidelines, case severity was categorized as mild, severe or critical. (*13*) A *mild* case was a patient with COVID-19 symptoms who was conscious and did not require oxygen support. A *severe* case was a symptomatic patient who was conscious but required oxygen support. A *critical* case was an unconscious patient either being treated with mechanical ventilation or receiving extracorporeal membrane oxygenation. Patients who had a chronic medical condition (e.g. cardiovascular disease, cancer, chronic respiratory diseases or diabetes) were defined as having pre-existing conditions at the time of infection.

Among confirmed cases, the status of being free from SARS-CoV-2 infection began on the date of the first of three consecutive negative SARS-CoV-2 tests before discharge. We used a sampling interval of 1 day between each test.

### Case finding and management

Cases were identified through PCR testing at the time of arrival in Viet Nam, during self-presentation at health facilities because of health concerns (due to travel history to regions recording confirmed cases) or through active case-finding measures among passengers and their contacts. All passengers on incoming flights from COVID-19 affected areas were tested for SARS-CoV-2 upon arrival and entered a mandated 14-day quarantine, irrespective of test results or symptoms. (The evolving test and quarantine policies for passengers arriving from affected areas into Viet Nam are included in **Supplementary  Table 1**.) During this period, passengers could leave the airport without testing or quarantine if they did not depart from defined designated areas, and passengers were only contacted when any co-passengers were confirmed to be positive for SARS-CoV-2.

Any person who presented to health facilities with symptoms compatible with COVID-19 and who reported a travel history to COVID-19 affected areas within the past 14 days was directly transferred to a reference hospital for SARS-CoV-2 testing and quarantine. Once SARS-CoV-2 infection was confirmed, an in-depth epidemiological investigation and contact tracing were conducted. All identified close contacts of confirmed cases were advised to self-quarantine immediately at their residence until contacted by local health authorities. They were then tested for SARS-CoV-2 by PCR and were placed into compulsory quarantine at a designated site for 14 days, irrespective of the test result. All quarantined individuals were tested at the start of their quarantine (day 0) and then systematically on days 3–5 and day 14. An additional test was undertaken if an individual developed symptoms. Anyone who tested positive or became symptomatic was transferred to a reference hospital for isolation and treatment.

### Statistical analysis

Data were cleaned using Microsoft Excel and exported to the statistical software package R version 3.6.3 for analysis. (*14*) Frequencies and percentages were used to describe the number of cases of each type (asymptomatic, pre-symptomatic and symptomatic), demographics and clinical symptoms in the study’s time range. Pearson’s χ^2^ and Fisher’s exact test were applied to compare demographic and clinical characteristics between re-positive and non-re-positive cases, and between cases with a negative test presenting with COVID-19 symptoms versus those without COVID-19 symptoms. The Kaplan–Meier estimator was used to assess time to clearance of SARS-CoV-2 infection; that is, the time between the date of symptom onset and the date of the first of three consecutive negative SARS-CoV-2 tests. Asymptomatic cases were excluded from this analysis. Log-rank tests were applied to explore the relationship between time to SARS-CoV-2 infection clearance and patients’ age, sex, pre-existing conditions, inconsistent PCR results and clinical severity.

## Results

Among the first 50 COVID-19 cases in the second wave in Viet Nam, the proportions of pre-symptomatic, symptomatic and asymptomatic cases were 38%, 42% and 20%, respectively. Male (54%) and female (46%) representation was approximately equal. Vietnamese nationals accounted for 64% of cases. The prevalence of people under 30 years old in pre-symptomatic, symptomatic and asymptomatic groups was 15.8%, 57.2% and 50%, respectively. Most of the asymptomatic and symptomatic cases were less likely than the pre-symptomatic cases to have a pre-existing condition (**Table 1**).

**Table 1:** Characteristics of COVID-19 cases by symptomatic category (*n* = 50)

-	Total		Pre-symptomatic		Symptomatic		Asymptomatic	
	*n* = 50	%	*n* = 19	%	*n* = 21	%	*n* = 10	%
Age, mean (SD)	40.6 (19.2)		48.6 (18.2)		35.1 (16.7)		36.7 (22.5)	
< 20	4	8	2	10.5	1	4.8	1	10
20–29	16	32	1	5.3	11	52.4	4	40
30–39	8	16	2	10.5	4	19	2	20
40–49	3	6	3	15.8	0	0	0	0
50–59	8	16	5	26.3	2	9.5	1	10
60–69	7	14	5	26.3	2	9.5	0	0
70+	4	8	1	5.3	1	4.8	2	20
**Sex**								
Male	27	54	12	63.2	11	52.4	4	40
Female	23	46	7	36.8	10	47.6	6	60
**Nationality**								
Vietnamese	32	64	10	52.6	15	71.4	7	70
Other	18	36	9	47.4	6	28.6	3	30
**Pre-existing condition**								
Yes	12	24	6	31.6	4	19	2	20
No	38	76	13	68.4	17	81	8	80

SD: standard deviation.

Two thirds of cases (*n* = 34, 68%) were international arrivals, with the remaining cases identified locally (*n* = 16, 32%). Among international passengers, 23% (*n* = 8) were detected through airport screening, 56% (*n* = 19) were detected through case-finding activities among flight passengers and 21% (*n* = 7) were detected during self-presentation at health facilities. All 16 local cases were close contacts of international passengers and were detected by case-finding activities (**Supplementary Fig. 1**).

**Supplementary Table 2** illustrates symptoms at onset and total numbers of symptoms during infection (combining symptoms at onset and during treatment or isolation) for the 40 pre-symptomatic and symptomatic cases. The most common symptom at onset was cough (70%), followed by fever (25%) and sputum production (15%). Most cases experienced multiple symptoms, with 70% having more than one symptom and 15% having six or more symptoms.

**Figure 1:**
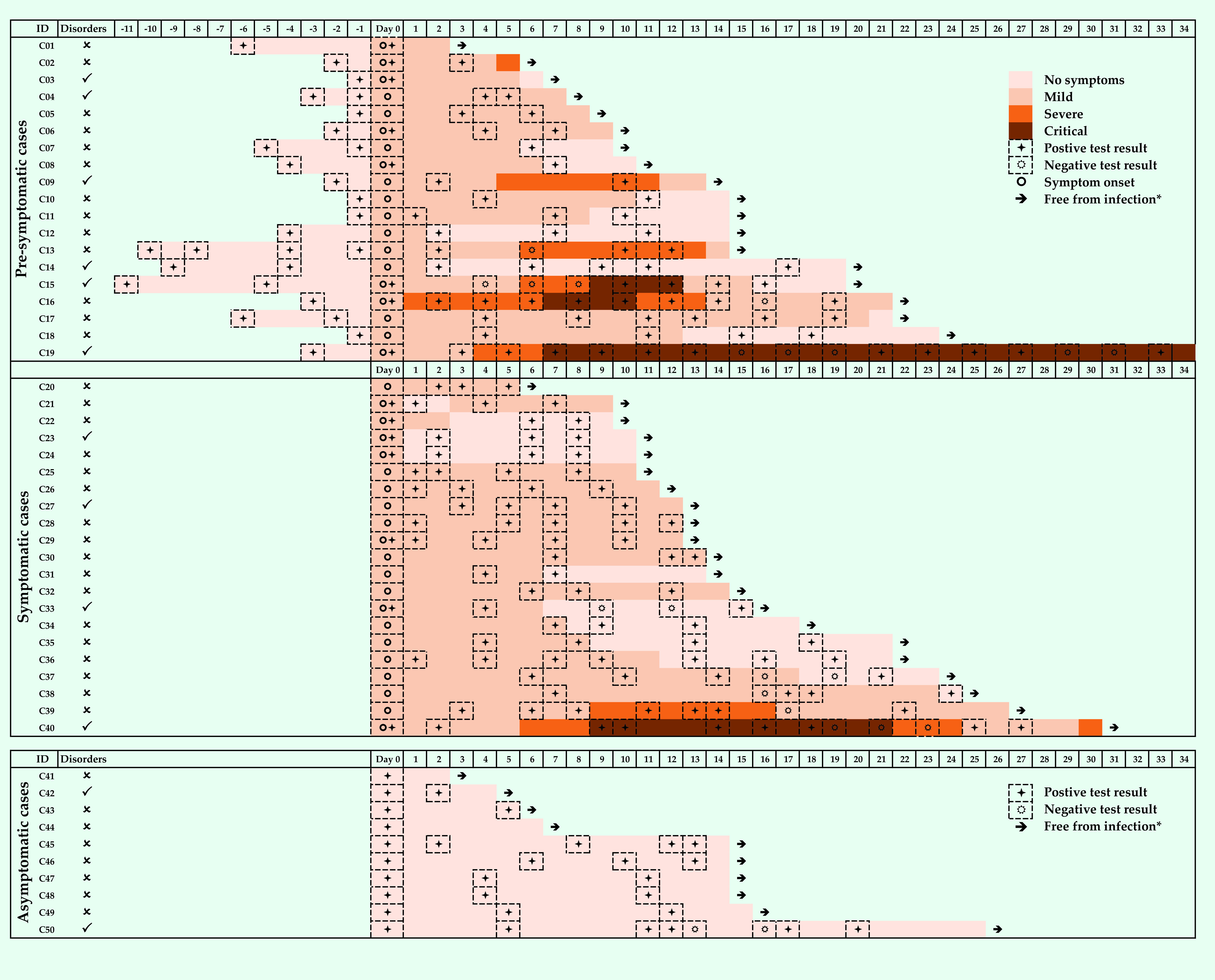
The evolution of clinical symptoms and results of SARS-CoV-2 PCR tests during treatment of symptomatic, pre-symptomatic and asymptomatic cases in Viet Nam

**Fig. 1** presents the clinical evolution and PCR results of SARS-CoV-2 testing during treatment or isolation in symptomatic, pre-symptomatic and asymptomatic cases. Among 40 (80%) patients who experienced symptoms during infection, eight (20%) were clinically classified as severe and four (10%) as critical. Three of the four critical cases had pre-existing conditions, namely, vestibular disorder, type 2 diabetes and hypertension.

[**insert** Figure 1]

Ten cases (20%) returned a positive SARS-CoV-2 result after returning one or more negative result (re-positivity). The number of re-positive cases who were pre-symptomatic, symptomatic and asymptomatic was four, five and one, respectively. Most re-positive cases (90%) had one loop of reversion (i.e. one positive test after a negative test, then negative test results until being free from SARS-CoV-2). Only one re-positive case (case 19, who was pre-symptomatic at the time of testing) had more than one loop of reversion.

Eight cases (16%) still experienced COVID-19 symptoms after testing negative for SARS-CoV-2, among which four (50%) were symptomatic and four (50%) were pre-symptomatic at the time of testing.

The demographic and clinical characteristics of the 10 (20%) re-positive COVID-19 cases and eight (16%) cases who had COVID-19 symptoms after testing negative for SARS-CoV-2 are shown in **Table 2**. There was no significant difference in age, sex, nationality, pre-existing conditions and symptoms at onset between re-positive and non-re-positive cases. This was also observed among symptomatic cases who had a negative test versus cases without symptoms. Most re-positive cases and cases with COVID-19 symptoms after testing negative were categorized as severe or critical, and experienced more than two symptoms during infection.

**Table 2:** Characteristics of COVID-19 cases with a re-positive PCR test and symptomatic cases with a negative PCR test (*n* = 50)

Characteristics	Re-positive test		*P^…^*	Negative test with COVID-19 symptoms		*P^…^*
	No *n*(%)	Yes *n*(%)		No *n*(%)	Yes *n*(%)	
**Demographics**						
Age						
< 45	26 (65)	3 (30)	0.10	28 (66.7)	1 (12.5)	0.01
45–64	8 (20)	5 (50)	-	8 (19)	5 (62.5)	-
65+	6 (15)	2 (20)	-	6 (14.3)	2 (25)	-
Sex						
Male	23 (57)	4 (40)	0.32	24 (57.1)	3 (37.5)	0.31
Female	17 (43)	6 (60)	-	18 (42.9)	5 (62.5)	-
Nationality						
Vietnamese	25 (63)	7 (70)	0.66	27 (64.3)	5 (62.5)	0.92
Other	15 (38)	3 (30)	-	15 (35.7)	3 (37.5)	-
Pre-existing condition						
Yes	7 (18.5)	5 (50)	0.03	9 (21.4)	3 (37.5)	0.33
No	33 (82.5)	5 (50)	-	33 (78.6)	5 (62.5)	-
**Disease characteristics**						
Symptoms at onset						
Cough	23 (57.5)	5 (50)	–	23 (54.8)	5 (62.5)	–
Fever	7 (17.5)	3 (30)	-	7 (16.7)	3 (37.5)	-
Headache	4 (10)	0 (0)	-	4 (9.5)	0 (0)	-
Fatigue	4 (10)	0 (0)	-	4 (9.5)	0 (0)	-
Sputum production	3 (7.5)	2 (20)	-	3 (7.1)	2 (25)	-
Sore throat	2 (5)	2 (20)	-	3 (7.1)	1 (12.5)	-
Chill	0 (0)	1 (10)	-	0 (0)	1 (12.5)	-
Nasal congestion	0 (0)	1 (10)	-	1 (2.4)	0 (0)	-
Diarrhoea	0 (0)	1 (10)	-	0 (0)	1 (12.5)	-
Number of symptoms during infection						
1–2	24 (60)	6 (60)	1	26 (61.9)	4 (50)	0.53
> 2	16 (40)	4 (40)	-	16 (38.1)	4 (50)	-
Patient category						
Pre-symptomatic	15 (38)	4 (40)	0.66	15 (35.7)	4 (50)	0.3
Symptomatic	16 (40)	5 (50)	-	17 (40.5)	4 (50)	-
Asymptomatic	9 (23)	1(10)	-	10 (23.8)	0 (0)	-
Severity						
Asymptomatic	9 (23)	1 (10)	0.009	10 (23.8)	0 (0)	< 0.001
Mild	28 (70)	4 (40)	-	29 (69)	3 (37.5)	-
Severe	2 (5)	2 (20)	-	2 (4.8)	2 (25)	-
Critical	1 (3)	3 (30)	-	1 (2.4)	3 (37.5)	-

^…^ **Groups were compared using Pearson’s χ^2^ or Fisher’s exact test.**

The overall median duration from onset of symptoms to clearance of SARS-CoV-2 infection was 14 days (range: 6–31). Twenty days after symptom onset, 75% (30 cases) were free from SARS-CoV-2 infection (**Supplementary Fig. 2**).

**Figure 2:**
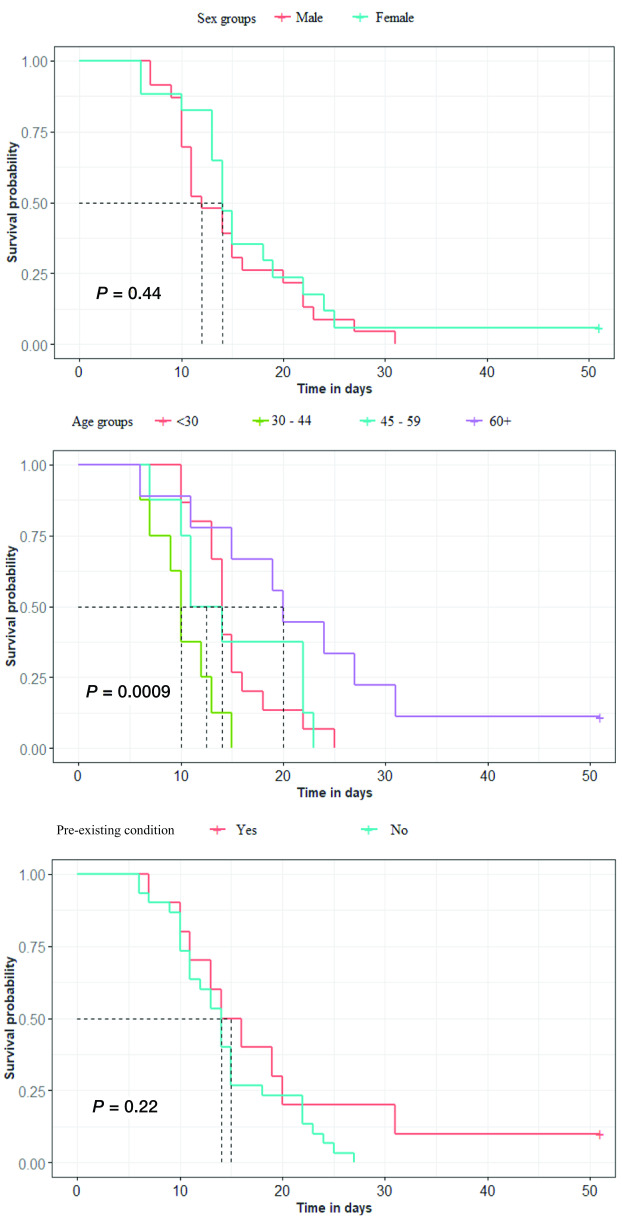
Cumulative probability by sex, age and pre-existing condition of clearance of SARS-CoV-2 infection from the day of first positive laboratory test among 40 pre-symptomatic and symptomatic cases

The median duration until clearance of SARS-CoV-2 infection was 12 days (95% confidence interval [CI]: 11–20) for males and 14 days (95% CI: 13–22) for females (*P* = 0.44), and was higher in older people (14 days among all those aged 30 years and older, 10 days in those aged 30–44 years, 12.5 days in those aged 45–59 years, 20 days in those aged 60 years or more; *P* < 0.001) (**Fig. 2**).

[**insert** Figure 2]

**Figure 3:**
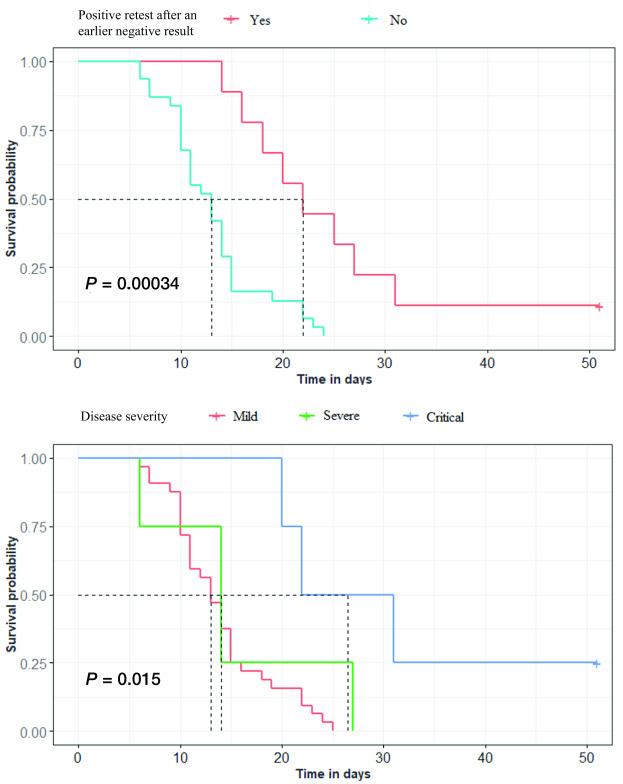
Survival analysis by re-positivity and severity of 40 pre-symptomatic and symptomatic cases

The duration until SARS-CoV-2 clearance for re-positive cases was nearly double the duration for those without test conversion (22 days vs 13 days, *P* = 0.00034). Critical cases had a longer time to freedom from infection (26.5 days) than did mild cases (13 days) and severe cases (14 days) (*P* = 0.015) (**Fig. 3**).

[**insert** Figure 3]

## Discussion

This study examined the clinical and laboratory findings of the first 50 SARS-CoV-2 confirmed cases at the start of the second COVID-19 wave in Viet Nam. There was a high prevalence of cases without compatible signs or symptoms of COVID-19 at the time of the first positive test (i.e. asymptomatic infections and pre-symptomatic infections). We found that 20% of cases tested positive following a negative result, and 16% of patients still experienced COVID-19 symptoms after testing negative for SARS-CoV-2. The median duration until clearance of SARS-CoV-2 infection was 14 days, with the duration being longer in older people, those with pre-existing conditions and re-positive cases.

The findings showed that 58% of cases did not exhibit compatible signs or symptoms of COVID-19 at the time of the first positive test, although fewer than half remained asymptomatic. This finding aligns with current published evidence of the expression of asymptomatic, symptomatic and pre-symptomatic cases. (*15*-*17*) The degree of SARS-CoV-2 transmission appears to vary among asymptomatic cases. A study in China showed that transmissibility from asymptomatic cases is comparable to that of symptomatic cases. (*18*) A study in Singapore suggested that people with asymptomatic COVID-19 might be less infectious than symptomatic cases. (*19*) Meanwhile, the World Health Organization declared that asymptomatic cases are much less likely to transmit the virus than those who develop symptoms. (*20*) However, there is still a lack of comprehensive studies with representative samples on SARS-CoV-2 transmission during the asymptomatic period.

In our study, 20% of confirmed COVID-19 cases returned a positive SARS-CoV-2 result after one or more negative test result. Findings from China indicated that the prevalence of a positive test following a negative test was about 17% after discharge. (*21*, *22*) Most current evidence about re-positivity focuses on the recovery or post-discharge phase. However, re-positivity during hospitalization might contribute to the need for ongoing admission and repeat testing, and cause distress for both patients and health care staff, which has not been the focus of published studies to date. In this study, all critical cases returned to positivity during SARS-CoV-2 infection. Re-positive cases had a substantially longer duration until viral clearance, which aligned with current evidence. (*21*)

Although re-positive tests for SARS-CoV-2 in recovered COVID-19 patients are common, there is insufficient evidence about the underlying mechanism leading to a re-positive test. (*23*) Most reported re-positive results could not be explained as simple viral relapse or secondary infection. (*24*) Some potential reasons included virology (biological characteristics of the virus), (*25*) specimen issues (sample collection, processing, virus at the limit of detection) (*26*-*28*) or patient condition (underlying conditions, degree of infection, treatment methods). (*29*) A study in post-symptomatic individuals showed that persistent positivity is associated with elevated cellular immune responses, and thus the viral RNA may represent replicating virus. (*30*) However, transmission to close contacts was not observed. Other evidence suggested that re-positive cases are not infectious after an initial negative test, indicating that persistent PCR-positive individuals are not infectious at the post-symptomatic stage of infection. (*11*, *31*) However, further work is needed to understand the likelihood of transmission from these patients.

Our findings showed that several cases still experienced COVID-19 compatible symptoms after testing negative for the virus or even after meeting SARS-CoV-2 clearance criteria. Defining and measuring COVID-19 transmissibility should be more sophisticated than only checking for a negative test. It has been suggested that when determining criteria for discharge and ending isolation, health authorities should consider multiple factors such as symptom resolution, time elapsed since the onset of symptoms, disease severity, immune system response and evidence of viral RNA clearance from the upper respiratory tract. (*32*)

Viral shedding is used as a marker of infectivity when detected via an upper respiratory tract PCR sample a few days before symptom onset. (*33*) Viral shedding persists for varying periods of time, with a median duration of 11 days.22 In our study, the median duration was 14 days. The viral shedding period in our study was defined as the day of diagnosis to the day of the first of three negative tests, each 24 hours apart; this excludes shedding before diagnosis. Although viral shedding has been identified during both the asymptomatic and symptomatic phases, its relation to transmissibility is unclear. Because real-time PCR cannot distinguish between infective virus and inactive virus, a positive PCR result does not necessarily represent the potential for viral transmission. The amount of viral RNA detected does not necessarily indicate greater infectivity.33

Older age and having pre-existing conditions have been reported as important independent predictors of worse outcomes in severe acute respiratory syndrome and Middle East respiratory syndrome. (*34*) Our results also confirmed that increased age and pre-existing conditions were associated with longer SARS-CoV-2 infection in COVID-19 patients, which is consistent with other findings. (*35*) Further in-depth studies are encouraged to explore additional factors related to the duration of SARS-CoV-2 infection.

We acknowledge that there were several limitations to this study. First, the relatively small number of cases and specific context might limit the generalizability of our study findings. Second, we acknowledge the lack of cycle threshold (Ct) values (the number of cycles necessary to detect the virus by PCR). Ct is a semiquantitative value that categorizes the concentration of viral genetic material in a testing sample following PCR testing. This value indicates how much viral genetic material is in the sample: a low Ct indicates a high concentration of viral genetic material, which is typically associated with a high risk of infectivity and vice versa. Knowing this value might have helped us to understand re-positivity tests and to compare symptomatic, pre-symptomatic and asymptomatic cases over time. Although Ct is important, this single value depends on several factors, including the quantity of specific gene targets and reagent variability, and other factors that do not reflect a person’s infectivity in the absence of clinical context. (*36*) Large-scale, multicentre studies that include Ct values are required to explore the importance of this issue.

## Conclusion

A high proportion of asymptomatic and pre-symptomatic infections were evident in the first 50 confirmed cases during the second wave of COVID-19 in Viet Nam. In this study, re-positive cases were common during hospitalization and had a long duration of SARS-CoV-2 infection. High-quality longitudinal studies to explore the transmissibility of re-positive and asymptomatic COVID-19 patients are needed.
