# Supplementary material for: Re-positive testing, clinical evolution and clearance of infection: results from COVID-19 cases in isolation in Viet Nam
Source: Western Pac Surveill Response J. 2021 Dec 13;12(4):1–11. doi: 10.5365/wpsar.2021.12.4.857 (PMC8873913; doi:10.5365/wpsar.2021.12.4.857)
Supplement: Supplementary file 1 [file wpsar-12-857-s001.pdf]

Supplementary Table 1. **Public health interventions to prevent transmission from flights to Viet Nam, 2020**

| Starting date | Actions |
|---|---|
| 1 January | Temperature screening[a] for passengers from Hubei, China at all entry points (airport, sea, land) in Viet Nam |
| 6 March | Case finding and contact tracing for all passengers on flights with identified infected case(s)<br><br>Mandatory health declaration for all inbound passengers from international flights |
| 14 March | SARS-CoV-2 testing at arrival and quarantine for passengers from the United Kingdom of Great Britain and Northern Ireland and all 26 countries in the Schengen Area |
| 18 March | Expanded SARS-CoV-2 testing upon arrival and quarantine for passengers from the United States, selected South-East Asian countries[b] and Russia |
| 21 March | SARS-CoV-2 testing upon arrival and quarantine for all passengers from international flights regardless of place of departure |
| 28 March | All international flights halted |

[a] Including both forehead temperature measurement and heat imaging.

[b] Brunei Darussalam, Cambodia, Indonesia, Lao People's Democratic Republic, Malaysia, Myanmar, the Philippines, Singapore and Thailand.
