# Supplementary material for: Re-positive testing, clinical evolution and clearance of infection: results from COVID-19 cases in isolation in Viet Nam
Source: Western Pac Surveill Response J. 2021 Dec 13;12(4):1–11. doi: 10.5365/wpsar.2021.12.4.857 (PMC8873913; doi:10.5365/wpsar.2021.12.4.857)
Supplement: Supplementary file 2 [file wpsar-12-857-s003.pdf]

Supplementary Fig. 1. **Methods of case detection[a] of 50 cases in Viet Nam**

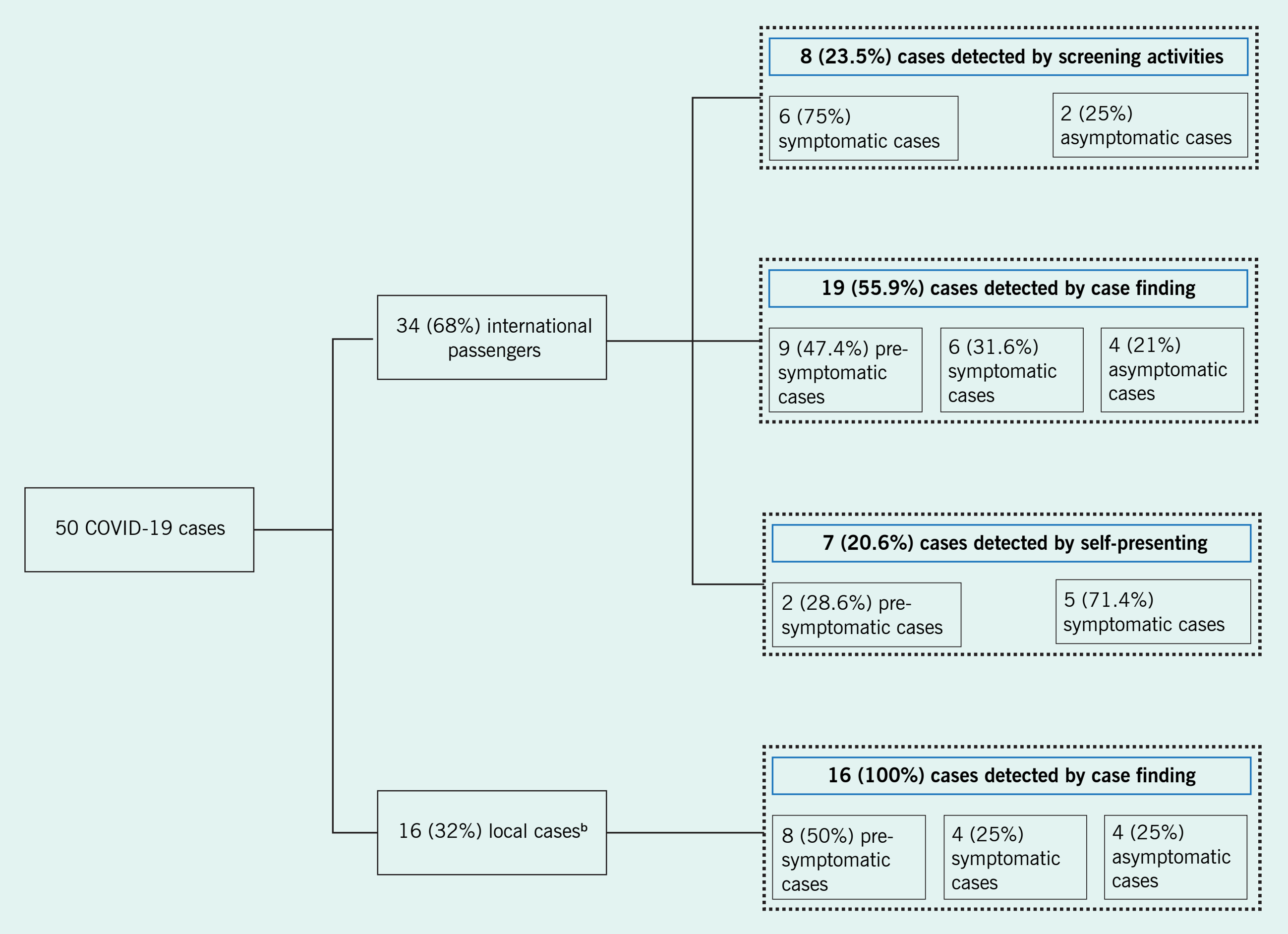

[a] Methods of case detection included: 1) screening activities (i.e. mandatory testing and quarantine immediately upon international arrival); 2) case finding (i.e. epidemiological investigations); and 3) self-presenting at health facilities.

[b] All 16 local cases were close contacts of the 34 international passengers.
