# Supplementary material for: Re-positive testing, clinical evolution and clearance of infection: results from COVID-19 cases in isolation in Viet Nam
Source: Western Pac Surveill Response J. 2021 Dec 13;12(4):1–11. doi: 10.5365/wpsar.2021.12.4.857 (PMC8873913; doi:10.5365/wpsar.2021.12.4.857)
Supplement: Supplementary file 3 [file wpsar-12-857-s002.pdf]

Supplementary Table 2. **Symptoms at onset and number of symptoms during infection[a] of 40 pre-symptomatic and symptomatic cases**

	n	%
Symptoms at onset[b]		
Cough	28	70
Fever	10	25
Sputum production	5	12.5
Sore throat	4	10
Headache	4	10
Fatigue	3	7.5
Nasal congestion	1	2.5
Diarrhoea	1	2.5
Chill	1	2.5
Total number of symptoms during infection		
1	12	30
2	8	20
3	8	20
4	3	7.5
5	3	7.5
6+	6	15

[a] Combined symptoms at onset and during treatment/isolation.

[b] Cases could have more than one symptom.
