# Supplementary material for: Re-positive testing, clinical evolution and clearance of infection: results from COVID-19 cases in isolation in Viet Nam
Source: Western Pac Surveill Response J. 2021 Dec 13;12(4):1–11. doi: 10.5365/wpsar.2021.12.4.857 (PMC8873913; doi:10.5365/wpsar.2021.12.4.857)
Supplement: Supplementary file 4 [file wpsar-12-857-s004.pdf]

Supplementary Fig. 2. **Cumulative probability of clearance of SARS-CoV-2 infection by time in days from symptom onset for 40 pre-symptomatic and symptomatic cases**

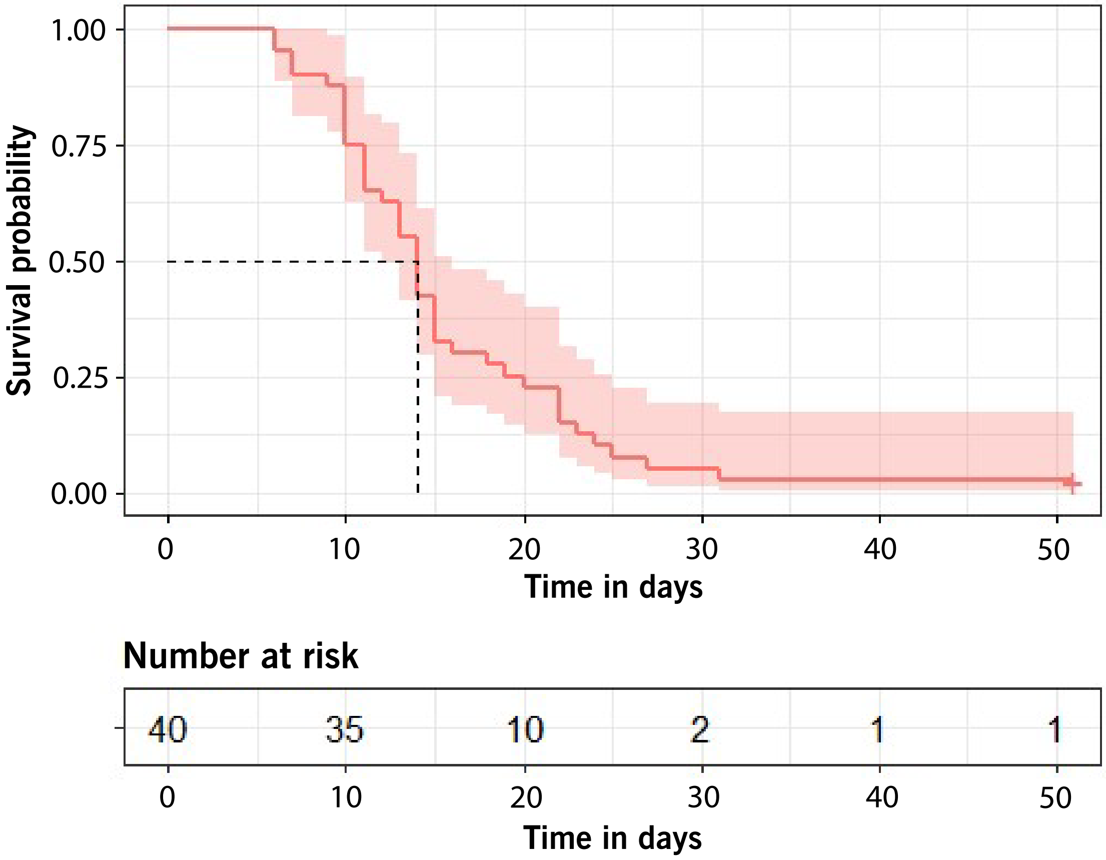

The survival probability was estimated using the Kaplan-Meier estimator.
